# Genome Mining Reveals Rifamycin Biosynthesis in a Taklamakan Desert Actinomycete

**DOI:** 10.3390/microorganisms13051068

**Published:** 2025-05-03

**Authors:** Xinrong Luo, Zhanwen Liu, Xiaoxia Luo, Zhanfeng Xia, Chuanxing Wan, Haoxin Wang, Lili Zhang

**Affiliations:** 1 State Key Laboratory Incubation Base for Conservation and Utilization of Bio-Resource in Tarim Basin, Tarim University, Alar 843300, China; luoxinrong365@163.com (X.L.); zwzky@163.com (Z.L.); xxluo415@163.com (X.L.); fenge3721@163.com (Z.X.); wanchuanxing@163.com (C.W.); 2 College of Life Science and Technology, Tarim University, Alar 843300, China; 3 State Key Laboratory of Microbial Technology, Shandong University, Qingdao 266237, China; wanghaoxin@sdu.edu.cn

**Keywords:** genome mining, *Actinomadura*, overexpression, rifamycin, Taklamakan Desert

## Abstract

Actinomycetes are recognized for producing diverse bioactive natural products, yet most biosynthetic gene clusters (BGCs) remain inactive under laboratory conditions. Rare actinomycetes from extreme environments represent underexplored reservoirs of metabolic potential. This study investigates *Actinomadura* sp. TRM71106, a rare actinomycete isolated from the Taklamakan Desert, through integrated genomic and metabolomic approaches. Genome sequencing revealed 45 secondary metabolic BGCs, including BGC38 showing 65% nucleotide similarity to the rifamycin BGC. Gene cluster networking and linear comparisons predicted its capacity to encode novel rifamycin analogs. Targeted activation strategies—overexpression of the pathway-specific regulator *LuxR* combined with metabolite isolation—mark the first activation of a rifamycin-like BGC in desert actinomycetes. This study highlights the untapped biosynthetic potential of rare actinomycetes in extreme environments and establishes *Actinomadura* sp. TRM71106 as a novel source for rifamycin production. These results provide a promising avenue for expanding the clinical pipeline of rifamycin-derived antibiotics.

## 1. Introduction

Microbial secondary metabolites are an important source of lead compounds, with 60% of anticancer drugs and 70% of antibiotics in clinical use derived from natural products and their derivatives [1]. Actinomycetes are a prolific source of antibiotics. Renowned for their extensive secondary metabolism, these microorganisms produce two-thirds of clinically used antibiotics [2], with *Streptomyces* sp. being the most significant contributor [3]. Although members of the genus *Streptomyces* remain the richest source of natural products, the likelihood of discovering novel antibiotics has diminished, shifting the research focus to rare actinomycetes [4]. Antibiotics from rare actinomycetes have diverse structures, unique activities, and significant developmental potential [5]. For instance, a rare *Mycobacterium* species produces asukamycin and apramycin [6], while *Amycolatopsis* species produce rifamycin [7].

Microorganisms from extreme environments have evolved unique defense and metabolic systems to survive adverse conditions, making actinomycetes from such habitats a promising focus for discovering new antibiotics [8]. These rare actinomycete resources have become a focal point of research globally [9]. The Taklamakan Desert, characterized by sparse rainfall, prolonged sunshine, intense evaporation, significant temperature fluctuations, and severe soil salinization [10], serves as a natural laboratory for microbial evolution [11]. Actinomycetes in this region harbor numerous secondary metabolite biosynthesis gene clusters (SM-BGCs) potentially encoding new natural products [12]. Notably, rare actinomycetes possess many unexpressed “silent biosynthetic gene clusters” [13], offering vast exploitation potential.

With the rapid advancement of sequencing technologies, especially third-generation sequencing [14], genomic information has become increasingly accessible. Analyses of sequenced data revealed that approximately 90% of biosynthetic gene clusters (BGCs) remain silent under standard laboratory growth conditions [15]. In the post-genomic era, the activation of these “silent metabolic pathways” through bioinformatics analysis to enhance the yield of encoded natural products has become a priority, leading to the generation of microbial secondary metabolites with enhanced structural and functional diversity for potential medicinal resource development [16].

Rifamycins are commercially significant antibiotics, with rifamycin B being a prominent example widely employed in the treatment of tuberculosis (TB), particularly drug-resistant *Mycobacterium tuberculosis* infections [17]. Because of their broad-spectrum antibacterial properties, they have also been repurposed for the treatment of other diseases [18]. These antibiotics eradicate bacteria by inhibiting RNA polymerases, thereby halting transcription and replication. However, the emergence of drug-resistant TB poses a global public health challenge [19], underscoring the need for ongoing efforts to identify and optimize new drugs. Consequently, research has focused on discovering novel rifamycins through combinatorial biosynthesis and gene editing techniques, boosting rifamycin production via genetic engineering, and exploring new bacterial sources for rifamycin production.

In this study, we conducted genome-wide analyses and annotation of *Actinomadura* sp. TRM71106, a rare actinomycete from the desert, using bioinformatics tools. The biosynthetic gene clusters (BGCs) of this strain were classified using the MIBiG database, facilitating the activation of novel silenced gene clusters via pathway-specific regulatory gene overexpression.

## 2. Materials and Methods

### 2.1. Genome Sequence Acquisition and Analysis

The collected organisms were precipitated, and the medium components were removed using sterile water. Total DNA was extracted using cetyltrimethylammonium bromide (CTAB). DNA concentration, quality, and integrity were assessed using a Qubit Fluorometer (Invitrogen, Carlsbad, CA, USA) and a NanoDrop Spectrophotometer (Thermo Scientific, Carlsbad, CA, USA). Sequencing libraries were prepared using a TruSeq DNA Sample Preparation Kit (Illumina, San Diego, CA, USA) and a Template Preparation Kit (Pacific Biosciences, Menlo Park, CA, USA). Genome sequencing was conducted by Personal Biotechnology (Shanghai, China) using the Nanopore PromethION48 and Illumina NovaSeq platforms. Adapter contamination and data filtering were performed using AdapterRemoval [20] and SOAPec [21], followed by assembly with SPAdes [22] and A5-miseq [23]. The final genome sequence was corrected using Pilon software [24].

Primers 27F and 1492R were utilized to amplify the 16S rRNA gene of strain TRM70308^T^. In addition, 16S rRNA-based phylogenetic trees were constructed using the Neighbor-Joining method with MEGA X [25]. The phylogenomic tree based on whole-genome sequences was generated using the Type (Strain) Genome Server platform (https://tygs.dsmz.de/, accessed on 8 December 2024.). GeneMarkS v4.32 [26] was used to predict the coding gene sequences. tRNA genes were predicted using tRNAscan-SE [27], while rRNA genes were identified using Barrnap v0.9. Furthermore, ncRNAs were predicted primarily through comparison with the Rfam database [28]. CRISPR Finder [29] was used to identify direct repeats and spacers in the genome. Gene evolutionary genealogy was predicted using Diamond 4 [30] based on the Non-supervised Orthologous Groups (eggNOG) database (http://eggnogdb.embl.de/#/app/home/, accessed on 2 December 2024.) to functionally annotate the predicted CDS. Genome sequences, gene functions, and non-coding RNA predictions were compiled into a standard GenBank (GBK) format file. A genomic circle map was plotted using cgview [31], which was subsequently edited using Photoshop CS.

To assess the biosynthetic potential of the strain, a complete contig was submitted with strict detection parameters to the antibiotics and Secondary Metabolite Analysis Shell (antiSMASH) web server (https://antismash.secondarymetabolites.org, accessed on 12 December 2024.) [32]. A local version of BiG-SCAPE (v1.1.5) [33] was used along with the antiSMASH and MIBiG databases, employing a distance cutoff score of 0.6. BGC networks from *Actinomadura* sequences were generated from these databases. The resulting clustered network graphs were refined and analyzed using Cytoscape 3.10.0 [34] and Clinker 0.0.27 [35] default algorithms. Visual covariance analysis of target gene clusters was performed using Easyfig (v2.2.5) software [36].

### 2.2. Strain, Plasmid, and Culture Conditions

TRM71106, isolated from the Taklamakan Desert and preserved by our group, and *Streptomyces* XZYN4 (containing the melC operon), isolated from Yunnan Province, China, and preserved at the State Key Laboratory of Microbial Technology, Shandong University, were used. *Actinomadura* sp. TRM71106 spore production was performed using Gauze’s (G1) solid medium. Tryptic soy broth served as the seed medium, while YMG medium was used for liquid fermentation. *Escherichia coli* DH5α and ET12567/pUZ8002, cultured in Luria–Bertani (LB) medium, were used for cloning and intergeneric conjugation, respectively, and plasmid pSET152 facilitated integration into TRM71106. All conjugants were cultivated on mannitol soybean meal (SFM) agar supplemented with 10 mM MgCl_2_. Apramycin (50 mg/mL) and nalidixic acid (40 mg/mL) were added to select the mutants. The media compositions are detailed in Table S1, and the strains and plasmids are listed in Table S2.

### 2.3. Construction of melC-Based Reporter Strains and the LuxR Overexpression Mutant Strain

The intp, SF14, and AACP promoter fragments and the melC operon from *Streptomyces* sp. XZYN4 were amplified using Phanta Max Superfidelity DNA Polymerase (Vazyme), and the primers are listed in Table S3. The *melC* operon was seamlessly cloned into the pSET152 integration vector using the In-Fusion HD Cloning Kit (Takara) under the control of each promoter to generate pSET152-intp-*melC*, pSET152-SF14-*melC*, and pSET152-AACP-*melC*. These constructs were introduced into *Actinomadura* sp. TRM71106 to create the recombinant strains TRM71106-IM, TRM71106-SM, and TRM71106-AM, respectively. These strains were cultured on MS medium containing 100 mg/L tyrosine and 0.5 mg/L Cu^2^⁺ to assess the expression of the *melC* operon.

*LuxR* (1.3 kb) was amplified using primers designed with high-fidelity Phanta Max Master Mix (Vazyme) and subsequently cloned into the pSET152-MELK plasmid at the *Nde*I and *EcoR*I sites downstream of the preferred promoter. This construct was introduced into TRM71106. Apramycin-sensitive mutants were selected after several rounds of screening, and their genotypes were verified via PCR amplification and sequencing, resulting in the successful acquisition of regulatory gene overexpression. The final mutant strain, TRM71106-IL, was confirmed using the M13F-47/RV-M primer pair. All primers utilized are listed in Table S3.

### 2.4. Fermentation, Isolation, and Analysis of Metabolites

Strains TRM71106 and TRM71106-IL were inoculated onto YMG agar and incubated at 30 °C for 12 d. Metabolites from solid cultures (50 mL) were extracted using EtOAc/MeOH/AcOH (80:15:5, *v*/*v*/*v*) and concentrated under reduced pressure and then dissolved in 0.5 mL MeOH. The extracts (30 μL) were analyzed via HPLC (Agilent 1260-ZORBAX Eclipse XDB-C18, 5 μm, 9.4 × 250 mm) with a gradient of 5–100% acetonitrile (solvent B) in water with 0.05% formic acid (solvent A) at 1 mL/min, with UV detection at 330 nm. Additionally, 5 μL was analyzed via LC-MS/MS (LTQ Velos Pro HRMS, Thermo Fisher Scientific, Shanghai, China ) under identical conditions.

TRM71106-IL was fermented on a 12 L scale in YMG solid medium, cut into small pieces, extracted three times with EtOAc/MeOH/AcOH (80:15:5, *v*/*v*/*v*), concentrated under reduced pressure, and further extracted three times with EtOAc/H_2_O. EtOAc crude extracts were separated using petroleum ether/MeOH. Following Sephadex LH-20 column chromatography (25–100 μm; Amersham Biosciences, Shanghai, China), five fractions were obtained. Fraction Fr.3 was enriched and further separated via medium-pressure liquid chromatography (MPLC) (200 g RP-18 silica gel, 40–63 μm; Merck KGaA, Darmstadt, Germany) using acetonitrile gradients (20%, 30%, 40%, 50%, 60%, and 100%), with 200 mL per gradient. Thirty percent of fraction Fr.1 was purified using silica gel (1 g, 200–300 mesh; Qingdao Haiyang Chemical Co., Ltd., Qingdao, China) and prepared via Thin-Layer Chromatography (TLC).

## 3. Results

### 3.1. Identification and Characterization of the TRM71106 Strain

In this study, the previously characterized strain TRM71106, which demonstrated activity against *Staphylococcus aureus* (ATCC 6538) from the soil of the Taklamakan Desert, was the focus of extensive research. The strain was characterized as Gram-positive, aerobic, and filamentous. Morphological observations of cultures grown on ISP-2 agar medium for 10 days revealed that aerial mycelium was scarce, and the strain exhibited robust growth on ISP-2, PDA medium. The basidiomycelium exhibited a color range from slightly yellowish to dark yellowish brown. However, the growth on ISP-3 and ISP-4 agar medium was only fair, with only very sparse white aerial mycelium observed after 14 days of incubation.

**Figure 1:**
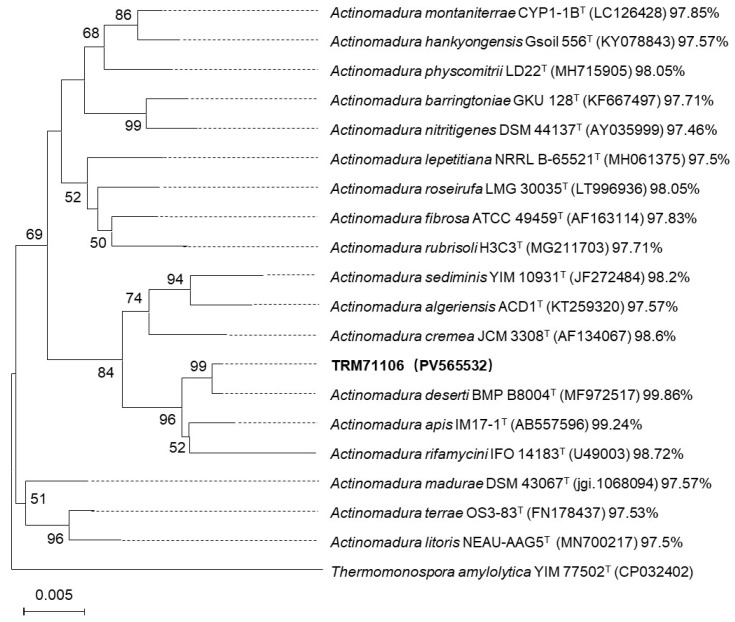
Phylogenetic tree of TRM71106 and related strains based on 16S rRNA gene sequences (NJ). Bootstrap values derived from 1000 replicates are shown at nodes (only values >50% displayed). The scale bar corresponds to 0.05 substitutions per nucleotide position. Accession numbers for 16S rRNA sequences are provided in brackets. Percentages indicate the similarity of TRM71106 to the respective strains. (T: type strains).

The 1441 bp 16S RNA gene sequence of TRM71106 exhibited a high degree of similarity to members of the genus *Actinomadura*. The phylogenetic analysis of TRM71106 was performed by sequencing the 16S rRNA gene. The sequence obtained was 1448 bp in length. Identification using the EZBioCloud server revealed that strain TRM71106 belongs to the genus *Actinomadura*, with the highest 16S rRNA gene sequence similarity to *A. deserti* BMP B8004T (99.86%), *A. apis* IM17-1T (99.24%), and *A. rifamycini* IFO 14183T (98.72%), which are the four strains that form a branch lineage with mutual bootstrap values greater than 50% (Figure 1).

**Figure 2:**
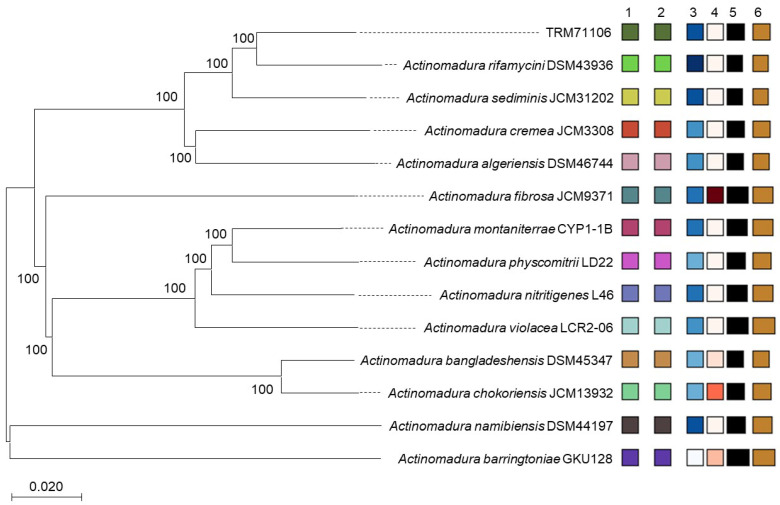
Phylogenomic tree of strain TRM71106 and related type strains, generated using the Type Strain Genome Server (TYGS). Numbers above branches represent genome blast distance phylogeny (GBDP) pseudo-bootstrap support values >60% (based on 1000 replicates). The tree was midpoint-rooted. Target strains are highlighted in bold. Key metrics: (1) species cluster, (2) subspecies cluster, (3) GC content (%), (4) Δ statistics, (5) genome size (bp), and (6) protein count (colors denote differences in indicator content).

Whole-genome sequencing data for *Actinomadura* sp. TRM71106 were organized, quality controlled, and assembled to yield a linear sequence of 8.98 Mb, with no linear or circular plasmids detected (Accession numbers: PRJNA1255387). A comprehensive genome-based reconstruction of the phylogenetic tree was conducted, which revealed that TRM71106 formed a branch lineage with *Actinomadura rifamycini* DSM43936 in the database, exhibiting a bootstrap value of 100% (Figure 2). As illustrated in the figure, significant disparities were observed in percent G+C, genome size, and protein count, while notable similarities were identified in species cluster. The genome size of TRM71106 was determined to be 8.9 Mb, and its GC content was found to be 73.31%.

The genome had a GC content of 73.31% and encoded 8208 genes. Of these, 7082 genes were annotated in the GOG database (Figure S1). The genome was found to contain 77 tRNAs and 15 rRNAs (comprising five 28S rRNAs, five 5S rRNAs, and five 16S rRNAs), along with nine CRISPR structures and 82 spacer regions. A genomic circle map was plotted using CGVIEW (Figure S2).

A comprehensive genomic analysis of strain TRM71106 revealed a variety of metabolic pathways, including glycolysis and gluconeogenesis, amino sugar and nucleotide sugar metabolism, glyoxylate and dicarboxylate metabolism, oxidative phosphorylation, glycine, serine, and threonine metabolism, propanoate metabolism, pentose phosphate pathway, citrate cycle (TCA cycle), pyruvate metabolism, and all of the genes for the enzymes of the major metabolic pathways.

Furthermore, we localized two sets of genes encoding the osmoprotectants betaine and tetrahydropyrimidine formation in the genome of this strain. These genes are included in the glycine, serine, and threonine metabolic pathways annotated using the KEGG. A set of betA genes (Ctg1_2454 and Ctg1_5585) were identified, which determine the enzymes for the oxidation of choline to betaine–aldehyde (choline dehydrogenase EC: 1.1.99.1) and its further conversion to betaine (betaine-aldehyde dehydrogenase, EC: 1.2.1.8), as well as genes for the glycine-betaine transporter system. Furthermore, a set of ectB genes were firmly identified (Ctg1_1841-1849), which determine tetrahydropyrimidine (ectoine) synthesis. The presence of these two sets of genes may be necessary for the strain to survive in such extreme environments as the desert.

### 3.2. Results of Biosynthetic Gene Cluster Analysis

Analysis of *Actinomadura* sp. TRM71106 using antiSMASH 7.0 revealed the presence of 45 BGCs (Table 1). Two clusters were experimentally verified to synthesize ectoine and 2-methylisoborneol, matching the MIBiG database [37]. Additionally, one BGC sharing 63% similarity with the known rifamorpholine BGC was identified. The remaining 42 BGCs were found to potentially encode novel natural products, with 32 BGCs displaying ≤50% category similarity to known clusters, while 10 BGCs exhibited no significant similarity to any currently known BGCs. Our results suggest that approximately 1.7 Mb of the TRM71106 genome is dedicated to natural product biosynthesis, accounting for 19.3% of the entire genome.

**Figure 3:**
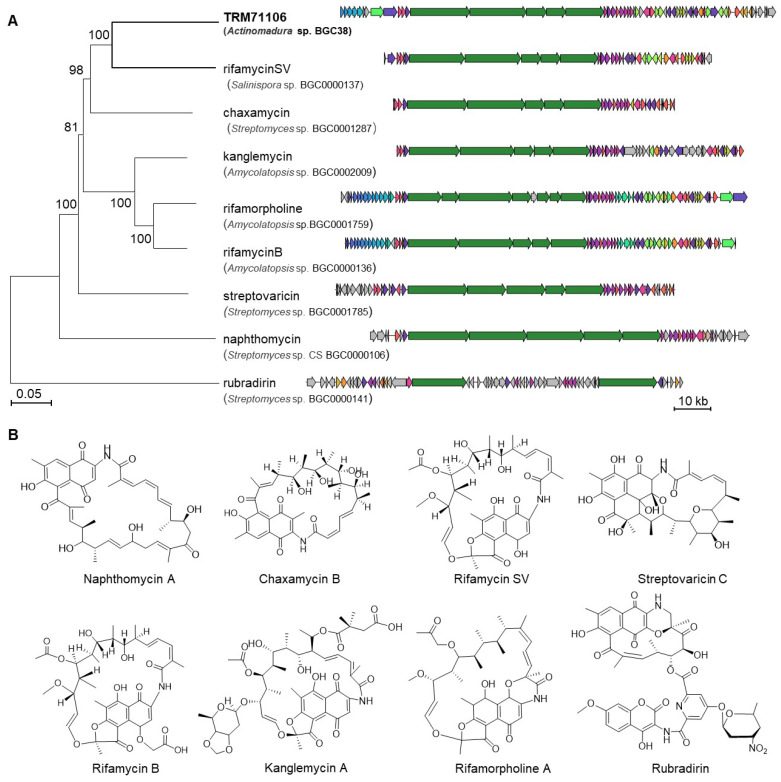
Genome mining analysis of *Actinomadura* sp. TRM71106. (**A**) Clustering of BGC38 with the MIBiG database of valid similar sequences and corresponding known natural product structures. The gene cluster from strain TRM71106 in this study is indicated in bold font, while the remaining clusters are sourced from the MIBiG database. (**B**) Validated compound structures clustered with BGC38 from the MIBIG database BGC codes.

Comparison and visualization of each BGC from *Actinomadura* sp. TRM71106 with known gene clusters in the MIBiG database revealed that BGC38 aligns with a group of eight biosynthetic genes encoding naphthalenic ansamycins, including naphthamycin, chaxamycin, rifamycin SV, streptovaricin, rifamycin B, kanglemycin, rifamorpholine, and rubradirin (Figure 3A). These compounds are characterized by a naphthalene nucleus connected to the polyketide chain via an amide bond at a nonadjacent position, forming a macrocyclic lactam structure and exhibiting a wide range of biological activities (Figure 3B). Although this gene cluster shares evolutionary relationships with other members, it exhibits significant phylogenetic divergence from its closest relative, the rifamycin SV cluster in the MIBiG database. This evolutionary distance highlights its potential to synthesize novel rifamycin-like compounds through divergent post-PKS tailoring pathways.

Further analysis of the 122 kb BGC38 identified typical ansamycin genes: *rifG*–*rifN*, which are required for the synthesis of AHBA; *rifA*–*rifE*, PKS genes required for polyketide chain synthesis; *rif-AS*, an amide synthase gene responsible for cyclization; and rif-32, a conserved pathway-specific regulatory gene for naphthalenic ansamycins. Importantly, Rif32 belongs to the LuxR family (Large ATP-binding LuxR regulators), and the constitutive expression of LuxR-type regulators has been demonstrated to activate otherwise silent PKS clusters [38]. Additionally, many genes with unknown functions participate in the post-modification of polyketides. These features strongly suggest its potential to encode rifamycin-like compounds.

**Figure 4:**
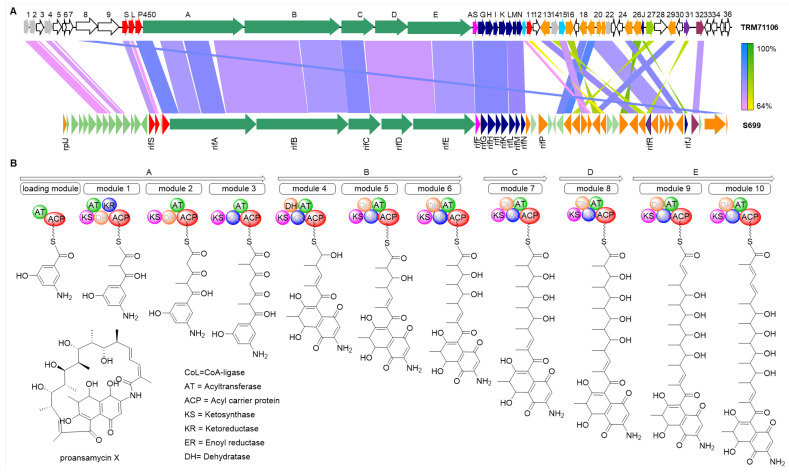
Comparative genomics and PKS architecture prediction of BGC38 in TRM71106. (**A**) Covariance analysis of TRM71106 and the rifamycin gene cluster. In the image, the top section displays BGC38 from *Actinomadura* sp. TRM71106, while the bottom section shows the rifamycin BGC from *Amycolatopsis* sp. S699. Genes with the same function are indicated by the same color, whereas white denotes genes with unknown functions. Lines connecting sections indicate genes with similar functions, and lines of different shades indicate the degree of function of the genes. (**B**) Predicted organisation of polyketide synthase from BGC38 in TRM71106 based on PKS domains.

A linear comparison analysis of BGC38 isolated from *Actinomadura* sp. TRM71106 with the rifamycin gene cluster from *Amycolatopsis* sp. S699 (Accession Number: BGC0000136) revealed high conservation in PKS and AHBA (3-amino-5-hydroxybenzoic acid) synthase regions (Figure 4A). The PKS genes showed 82.1% identity, AHBA synthase 81.9%, and amide synthase 73.9% identity. The polyketide synthase organization in BGC38 exhibited similarity to that of rifamycin producers (Figure 4B) [39]; however, significant divergences in post-modification gene arrangement and sequence homology clearly demonstrate that TRM71106 produces novel compounds with rifamycin-like backbone structures (Figure 4).

### 3.3. TRM71160-IL Srain Construction and Detection

Given the clinical importance of rifamycins as last-resort drugs for multidrug-resistant tuberculosis, and the bioinformatic analysis in Section 3.2 showing that BGC38 has the capability to synthesize novel rifamycin analogues (Figure 4A), we explored the secondary metabolites of *Actinomadura* sp. TRM71106. Despite intensive chemical analysis using large-scale fermentation, no intact rifamycin-related metabolites were detected in the wild-type strain. To facilitate the production of rifamycin, we employed overexpression of pathway-specific regulators for ansamycin compounds. Initially, a melanin reporter gene was utilized to screen for highly active promoters. Three promoters, *intp*, *AACP*, and *SF14*, were amplified via PCR, and the *melC* operon was placed under their control to create the reporter plasmid. These constructs were then integrated into the strain TRM71106 to produce the reporter strains TRM71106-IM, TRM71106-SM, and TRM71106-AM. The YMG agar medium was used to assess metabolite production. When the three reporter strains were grown on the YMG agar medium, they exhibited varying degrees of black coloration, with TRM71106-IL showing the darkest color (Figure S3). This suggested that the *intp* promoter exhibited the highest activity under these culture conditions.

The LAL family regulatory gene *rif32* in the TRM71106 WT strain was expressed under the control of the *intp* promoter, resulting in the recombinant strain TRM71106-IL (Figure S4). After 10 d of incubation at 30 °C, small-scale fermentation and HPLC analysis of strain TRM71106-IL revealed multiple peaks absent in the control WT strain TRM71106, with remaining peaks showing a considerable yield increase, approximately three-fold that of the WT (Figure S4).

### 3.4. Rifamycin Produced by Strain TRM71160-IL

Despite optimizing the splicer, fermentation duration, medium, and precursor (AHBA) feeding, the mutant strain showed no significant yield differences in peak profiles. Strain TRM71106-IL was subjected to solid-state fermentation in 12 L of YMG medium at 30 °C for 12 d. Methanol extracts were obtained and further processed using MPLC, Sephadex LH-20, silica gel column chromatography, and TLC to isolate Compound 1. The remaining fractions could not be separated because of their low yields.

**Figure 5:**
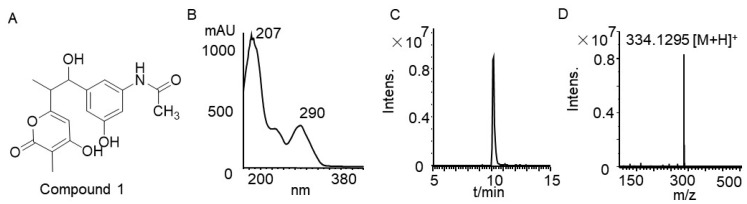
(**A**) Chemical structure of SY4b; (**B**) UV/Vis spectrum of SYB4; (**C**,**D**) ESI-HRMS spectrum of SY4b.

Compound **1** was isolated as a red powder that was soluble in methanol. Its molecular formula was determined as C_18_H_16_NO_6_ via electrospray ionization high-resolution mass spectrometry (ESI-HRMS) with HRESIMS *m*/*z* 334.1295 [M + H]^+^ (calcd. for 334.1291). Analysis of ^1^H-NMR and ^13^C-NMR spectra (Figures S5 and S6) and comparison with the literature identified the compound as SY4b (Figure 5), a linear shunt product of rifamycin [40].

SY4b exhibited a retention time of 9.8 min, followed by metabolites with similar UV absorption and differential peaks with retention times of 25–30 min (Figure S7). Rifamycin analog synthesis involves an assembly-line process for polyketide chain construction; disruptions can lead to linear precursors detectable via analysis. Longer retention times correlate with extended PKS chains and increased molecular weights [40]. Based on the characteristics of the rifamycin analogs, we hypothesized that this series of absorption peaks corresponds to rifamycin and its linear precursors. LC-MS/MS analysis of the regulatory gene-overexpressing mutant strain TRM71106-IL revealed a sequential mass profile aligned with retention times and incremental molecular weights: 320.1136 (calcd for C_16_H_17_NO_6_⁺), 344.1138 (calcd for C_17_H_20_NO_6_⁺), 402.1557 (calcd for C_21_H_23_NO_7_⁺), 442.1873 (calcd for C_24_H_27_NO_7_⁺), 518.2399 (calcd for C_27_H_35_NO_9_⁺), and 602.2976 (calcd for C_32_H_43_NO_10_⁺) (Figure S7). These masses matched previously reported rifamycin linear intermediates, namely SY4a, SY5, SY6, SY7, SY8, and SY10 [41]. Additionally, two masses detected at retention times of 26–27 min, 696.2659 (calcd for C_37_H_45_NO_12_⁺) and 726.2636 (calcd for C_38_H_47_NO_13_⁺), corresponded to the molecular weights of rifamycin S [42] and 3-formyl rifamycin [43], respectively. However, the exact structural identities of these compounds, particularly stereochemical configurations and post-PKS modifications, remain to be resolved through orthogonal techniques such as high-field NMR spectroscopy or MS/MS fragmentation analysis.

## 4. Discussion

Ongoing advancements in natural product biosynthesis have culminated in the refinement of genome-based natural product discovery technology. This bioinformatics-driven approach, coupled with an established compound screening system and systematic gene annotation and modification, significantly enhances the likelihood of discovering new compounds, thus making the search for natural products more targeted [44]. In this study, we sequenced the genome of the desert-derived actinomycete TRM71106, utilizing various public databases and software for gene prediction and protein function annotation. Our analysis suggested that this strain contains abundant BGCs, notably BGC38, which encodes a novel rifamycin-like compound. Rifamycin, the first member of the ansamycin family, was discovered by Sensi in 1959 [45]. Rifamycin B was introduced clinically in 1962 following chemical modification [46] and has been extensively used in the treatment of TB. Subsequent derivatives such as rifamycin SV, rifampicin, rifapentine, and rifabutin have become essential antibiotics in clinical practice [47]. Rifamycin is a critical polyketide with broad antimicrobial activity, no cross-resistance, and diverse biological functions. It features a macrocyclic structure formed by an aliphatic ansa chain linked at the nonadjacent positions of an aromatic naphthalene chromophore via a phthalamide bond, classifying it as a macrolide antibiotic [48]. The aliphatic chain length, if greater than 23 carbon atoms or less than 15 carbon atoms, remains stable, whereas some chains are interrupted by an oxygen atom, as observed in rifamycin. Additionally, the aliphatic chain may host a variety of substituents, with further possibilities for different substituents at specific positions in the aromatic nucleus [49]. Given that BGC38 encompasses numerous novel post-modification genes, many undiscovered rifamycin structures are predicted to exist, underscoring the importance of searching for new rifamycin analogs in drug development.

After overexpressing the pathway-specific regulatory gene *Lux*R in the BGC38 of strain TRM71106, we isolated a linear precursor of rifamycin, which was produced at approximately three times the yield of the wild-type strain. Although intact rifamycin could not be isolated, LC-MS analysis revealed its presence, suggesting that regulatory gene overexpression successfully activated the rifamycin-like gene clusters. This is the first time rifamycin has been detected as a metabolite of desert-sourced actinomycetes. While mass accuracy-guided annotation strongly supports the detection of rifamycin-like intermediates, it is critical to note that definitive structural validation requires orthogonal analyses. The low yield of rifamycin in this fermentation product was attributed to the accumulation of linear intermediates, likely due to incomplete activation of the *rifE* or *rifAS* genes within the cluster. Enhancing the expression of these two genes may increase the number of cyclic rifamycin analogs, leading to the discovery of novel rifamycins.

The expression of natural product gene clusters is regulated by complex interactions between various regulatory elements, with pathway-specific regulatory genes playing only a partial role. However, these approaches proved inadequate for rifamycin mining in TRM71106. Although many compounds of the same ansamycin-like series could be analyzed using HPLC, numerous components with lower contents could not be obtained in sufficient amounts or purity for spectroscopic determination and structural elucidation because of their low abundance during the isolation process. To improve this, we should consider optimizing the fermentation conditions, expanding the fermentation scale, and combining molecular networks [50], MicroED, and other technologies to obtain more structural information from trace amounts of novel ansamycins. Therefore, relying solely on this strategy may not always be effective and may require the assistance of other transcriptional regulatory elements, such as promoter insertions or substitutions. Promoter engineering by replacing native promoters with synthetic ones can significantly enhance gene transcription, activate clusters, and increase yield. Additionally, the knockout of negatively regulated genes can activate gene clusters [15]. Actinomycetes contain large genomes encoding diverse BGCs. Deactivating by-product-encoding clusters and eliminating complex metabolic backgrounds can effectively enhance the yield of target gene cluster products [51].

While heterologous promoters often exhibit host-dependent activity in actinomycetes, our screening of intP, AACP, and SF14 in *Actinomadura* sp. TRM71106 revealed that only intP robustly activated the melC reporter (Figure S3), implying that promoter compatibility in actinomycetes requires empirical validation. The success of intP in driving both melC expression and BGC38 activation (Figure S4) does not negate the observed inefficiency of other promoters; rather, it emphasizes the need for host-specific promoter engineering to activate silent clusters in extremophilic strains. Moreover, candidate strains have complex metabolic profiles that may interfere with the detection and isolation of target gene cluster products under conventional conditions. To address this, we will eliminate the complex metabolic backgrounds through multiple gene editing. Finally, the original strains used for genetic manipulation are inefficient, with prolonged transformation cycles. To overcome this, we performed large-scale cloning using a heterologous expression strategy in a model chassis bacterium for product mining. In future work, we will continue to activate the silent gene cluster in TRM71106 using various strategies to discover new natural products.

## 5. Conclusions

The genomic characterization of *Actinomadura* sp. TRM71106 reveals the biosynthetic potential of extremophilic actinomycetes, identifying 45 secondary metabolite biosynthetic gene clusters (BGCs). LuxR-mediated activation of BGC38 enabled the first detection of rifamycins in desert-derived actinomycetes, demonstrating the efficacy of regulator overexpression in silent cluster activation. These findings expand the ecological distribution of rifamycin producers, establish extremophilic microbes as priority targets for antibiotic discovery, and provide a methodological framework for exploring silent BGCs. The authors of future studies should optimize metabolite yields through fermentation engineering and investigate combinatorial activation strategies. This work highlights desert ecosystems as untapped reservoirs for antimicrobial discovery while advancing practical approaches for silent cluster activation.

## Figures and Tables

**Table 1:** Description and genomic positions of biosynthetic gene clusters (BGCs) in the complete linear genome of *Actinomadura deserti* TRM71106. NRP, non-ribosomal peptide synthetase; PKS, polyketide synthase; N, no significant similarity to any currently known BGCs.

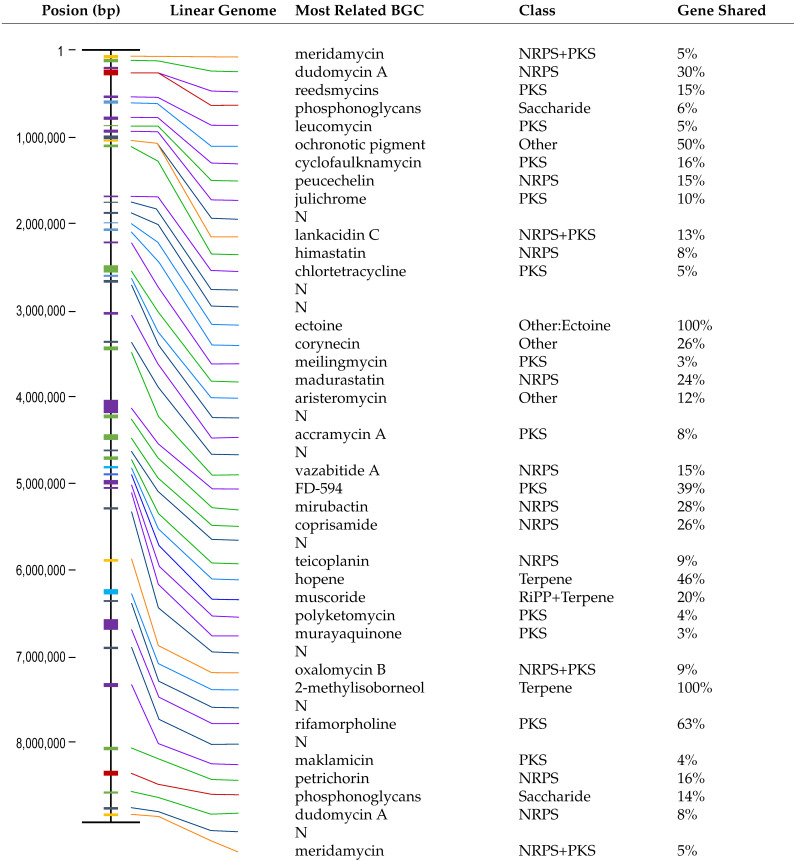				

## Data Availability

The GenBank accession number of the 16S rRNA gene sequences and BioProject number of draft genome of strain TRM71106 are PV565532 and PRJNA1255387, respectively; additionally, all other datasets used and analyzed during the current study are available from the corresponding author on reasonable request.

## Supplementary Materials

The following supporting information can be downloaded at https://www.mdpi.com/article/10.3390/microorganisms13051068/s1. Figure S1: *Actinomadura* sp. TRM71106 eggNOG (COG) functional classification chart; Figure S2: Genome Circle Diagram of TRM71106; Figure S3: Promoter screening using the melC reporter plasmid. IM, SM, and AM represent intp-melC, SF14-melC, and AACP-melC, respectively; Figure S4: Construction and functional validation of overexpression strain TRM71106-IL; Figure S5: 1HNMR spectrum of compound 1; Figure S6: 13CNMR spectrum of compound 1; Figure S7: HPLC analysis and HRMS mass map of the TRM71106-IL metabolite. Table S1: General media used in this study; Table S2: Strains and plasmids used in this study; Table S3: Primers used in this study. Refs. [52,53] are cited in the Supplementary Materials.
