# Supplementary material for: Genome Mining Reveals Rifamycin Biosynthesis in a Taklamakan Desert Actinomycete
Source: Microorganisms. 2025 May 3;13(5):1068. doi: 10.3390/microorganisms13051068 (PMC12114206; doi:10.3390/microorganisms13051068)
Supplement: Supplementary file 1 [file microorganisms-13-01068-s001.zip › microorganisms-3585234-supplementary.pdf]

Table S1. General media used in this study

medium	
Gauze's (G1)	20 g/L soluble starch, 0.5 g/L K_2HPO_4, 0.5 g/L NaCl, 1 g/L KNO_3, 0.5 g/L $MgSO_4 \cdot 7H_2O$ and 0.01 g/L $FeSO_4 \cdot 7H_2O$, (pH 7.0)
Tryptic soy broth (TSB)	17 g/L tryptone, 3 g/L soy peptone, 2.5 g/L K_2HPO_4, 5 g/L NaCl and 2.5 g/L glucose (pH 7.0)
Luria-Bertani (LB)	10 g/L tryptone, 5 g/L yeast extract, and 10 g/L NaCl; for solid medium 15 g/L agar was added (pH 7.0).
Mannitol soya flour (MS)	20 g/L mannitol, 20 g/L soybean flour, and 15 g/L agar (pH 7.0).
Yeast-Malt-Glucose (YMG)	(yeast extract 4.0 g, malt extract 10.0 g, glucose 4.0 g, agar 15.0 g, distilled water 1.0 L, pH 7.4)

Table S2. Strains and plasmids used in this study

Strains/plasmids	Descriptions	Reference/Source
E. coli strains		
DH5α	General cloning	Commercial source
ET12567/pUZ8002	Methylation defective, strain used for *E. coli-Streptomyces* intergeneric conjugation	(Kieser T, 2000)
Streptomyces strains		
TRM71106	*Actinomadura* sp. TRM71106 wild type	This study
TRM71106-IL	Containing plasmid pSET152-intp-LAL	This study
Plasmids		
pSET152	*E. coli* vector, *phi*C31 Integrative in *Streptomyces*, Apr^R	(Bierman M,1992)
pSET152-intp-MELC	int promoter-driven melC1-C2-neo, as a promoter activity reporter	This study
pSET152-AACP-MELC	AACP promoter-driven melC1-C2-neo, as a promoter activity reporter	This study
pSET152-SF14-MELC	SF14 promoter-driven melC1-C2-neo, as a promoter activity reporter	This study
pSET152-intp-LAL	pSET152 derivative containing intp-rif32	This study

Table S3. Primers used in this study

Primer	The nucleotide sequence (5'-3')	Function
152 intpM-F	CAGGTCGACTCTAGTATGCATGCCGGAG CAATCGCC	Amplifying the intp promoter
152 intpM-R	ACGACTGAGTTCCGGCATAGCCGCGGT CACAAACCC	
152 SF14M-F	GAGCCATGACTGCAGCCA TGGTGCTTTGCGATACCGACCACC	Amplifying the SF14 promoter
152 SF14M-R	AGGCCACGAGTTCGGTCTAGATGTGCG CCCACATCCCTC	
152 AACPM-F	CCGAACTCGTGGCCTCCTCA	Amplifying the AACP promoter
152 AACPM-R	CCGCGTGCACTTCGAAGCTTGCGGTGT GTGTCCTCTGCTC	
71106-LUX-F	GACCGGGCATATGCAATCTTCTCTGGGT TCCCTCAC	Amplifying the LuxR in *Actinomadura* sp. TRM71106
71106-LUX-R	TACGAATTCTTACGGGTTCGCCGCCCAG	
M13-47	CGCCAGGGTTTTCCCAGTCACGAC	PCR confirmation of TRM71106-IL mutant
RV-M	TGTGTGAAATTGTTATCCGCT	

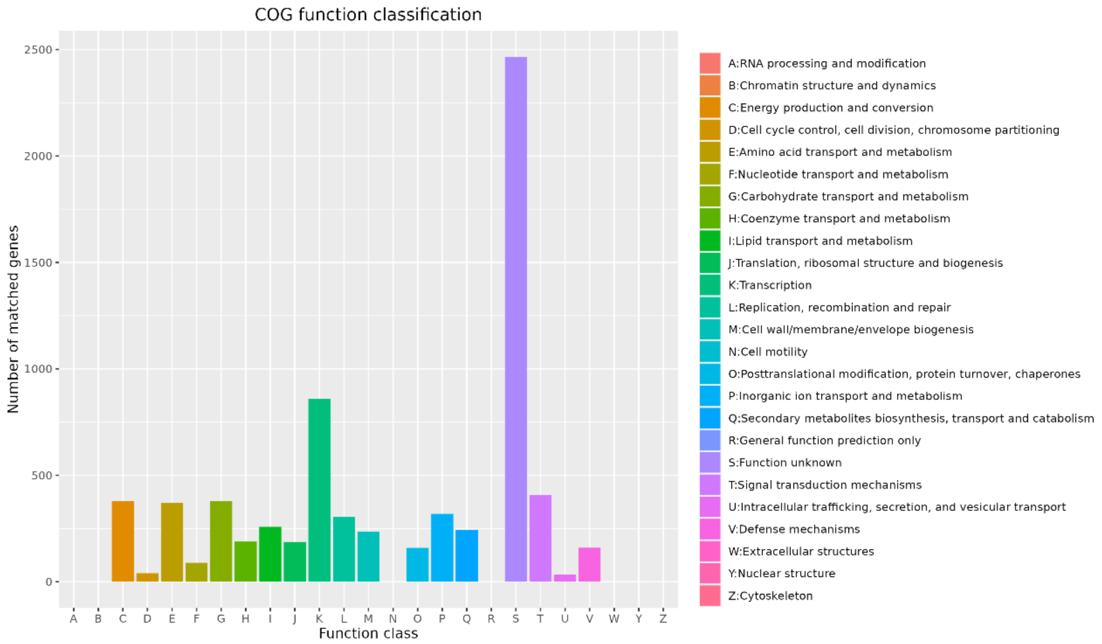

Figure S1. Actinomadura deserti TRM71106 eggNOG (COG) Functional Classification Chart

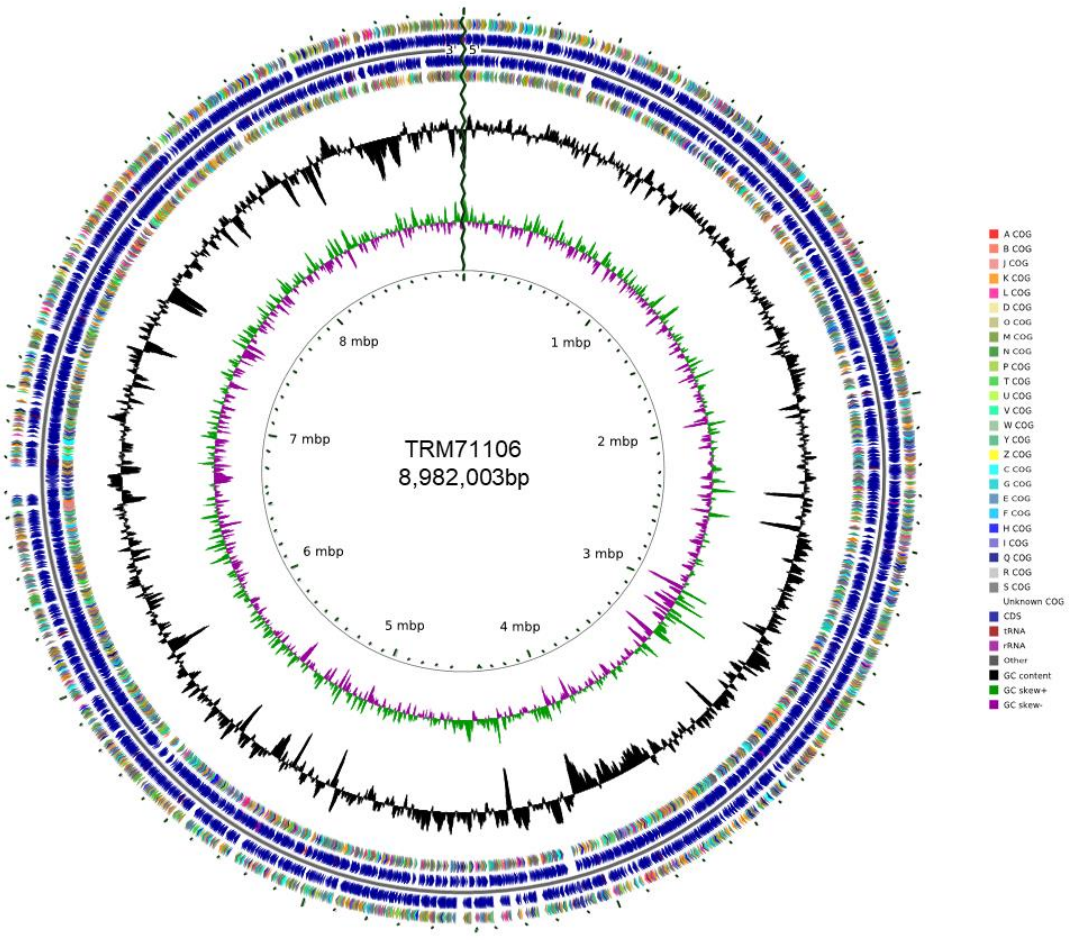

Figure S2. Genome Circle Diagram of TRM71106. The circular representation of the TRM71106 genome displays various genomic features from the innermost to the outermost circles. The diagram includes: (1) the scale of the genome, (2) the GC skew, (3) the GC content, (4) and (7) the COG categories for each CDS, and (5) and (6) the spatial distribution of CDSs, tRNAs, and rRNAs.

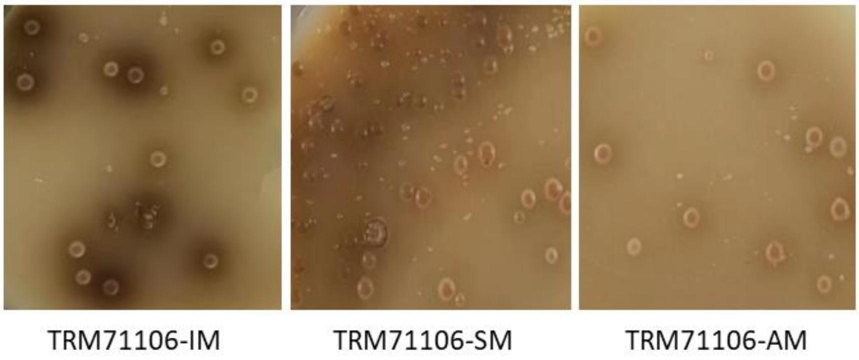

Figure S3. The promoter screening through melC reporter plasmid. IM, SM, AM represent intp-melC, SF14-melC, AACP-melC, respectively. The darkening effects reflect temporal promoter activity under specific cultivation conditions (YMG agar, Day 10) and may not represent universal expression levels.

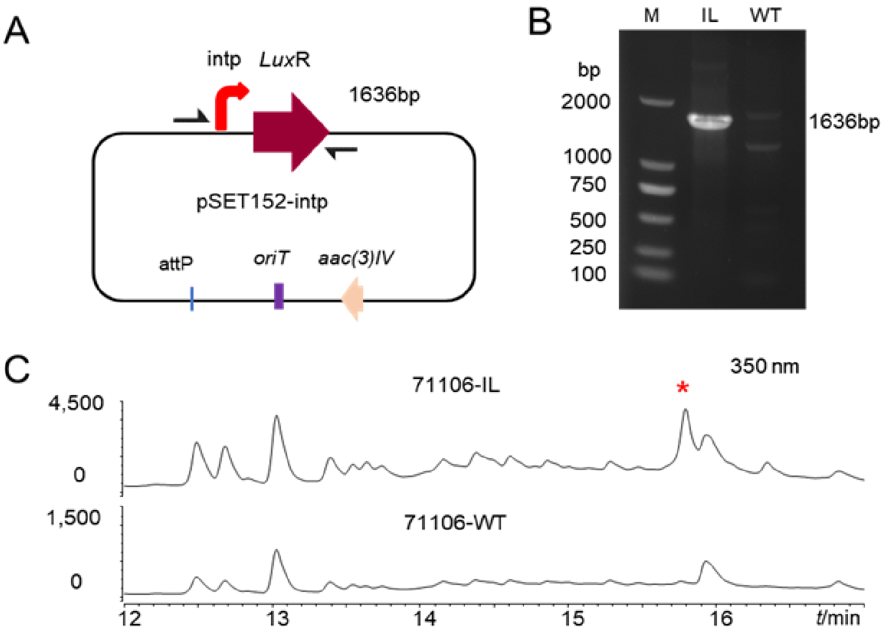

Figure S4. Construction and functional validation of overexpression strain TRM71106-IL. (A) pSET152-TRM711JH-IL plasmid construction; (B) Agarose gel electrophoresis analysis of PCR validation for the TRM71106-IL mutant. Lanes: M, DNA ladder; WT, wild-type strain; IL, mutant strain. (C)HPLC analysis of the fermentation products of TRM71106-WT and TRM71106-IL on YMG medium

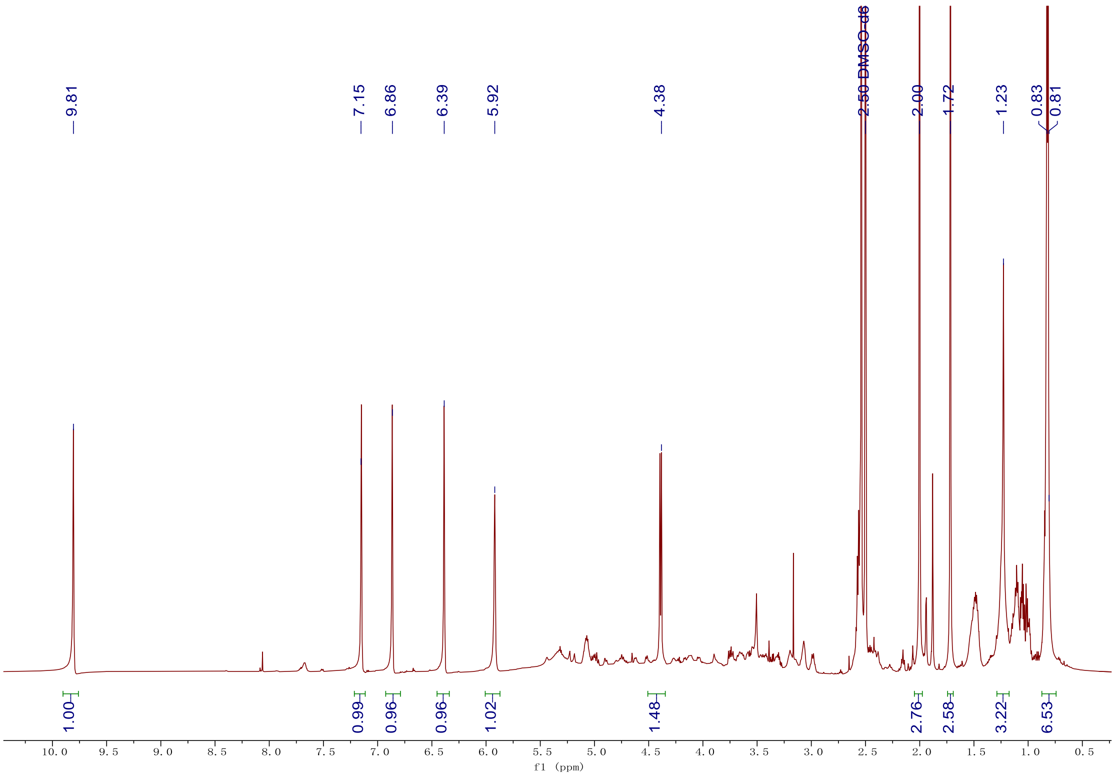

Figure S5. ^{1}H NMR spectrum for compound **1**

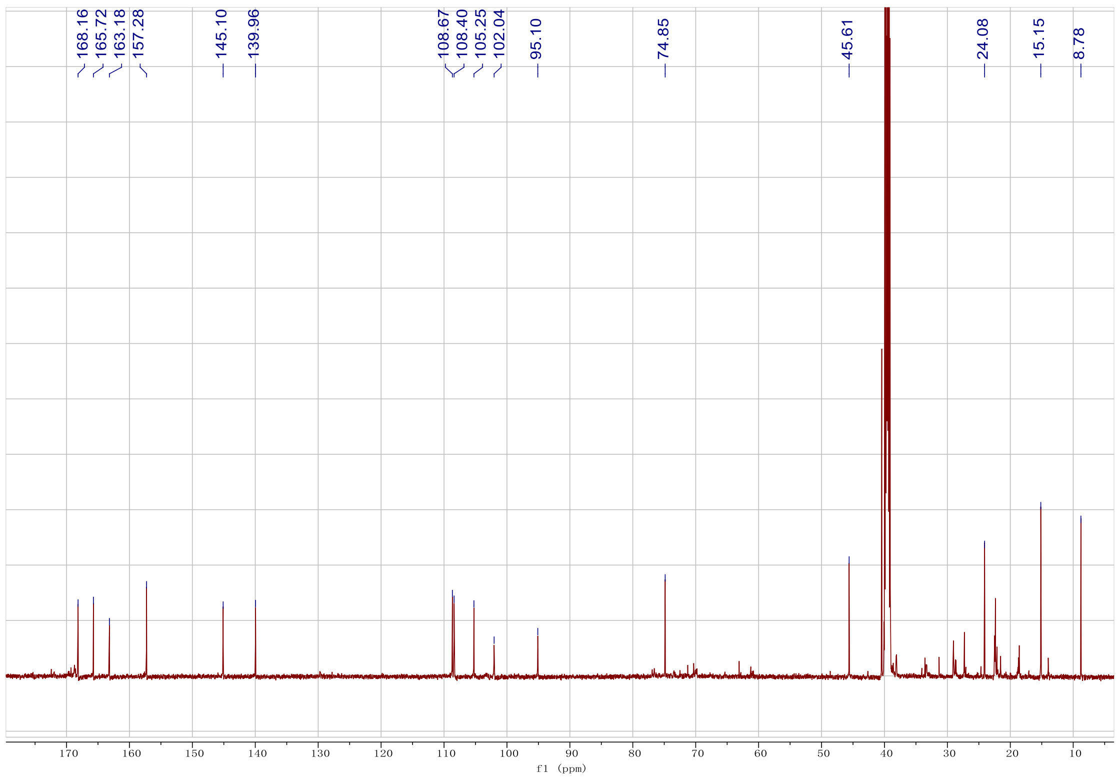

Figure S6. ^{13}C NMR spectrum for compound **1**

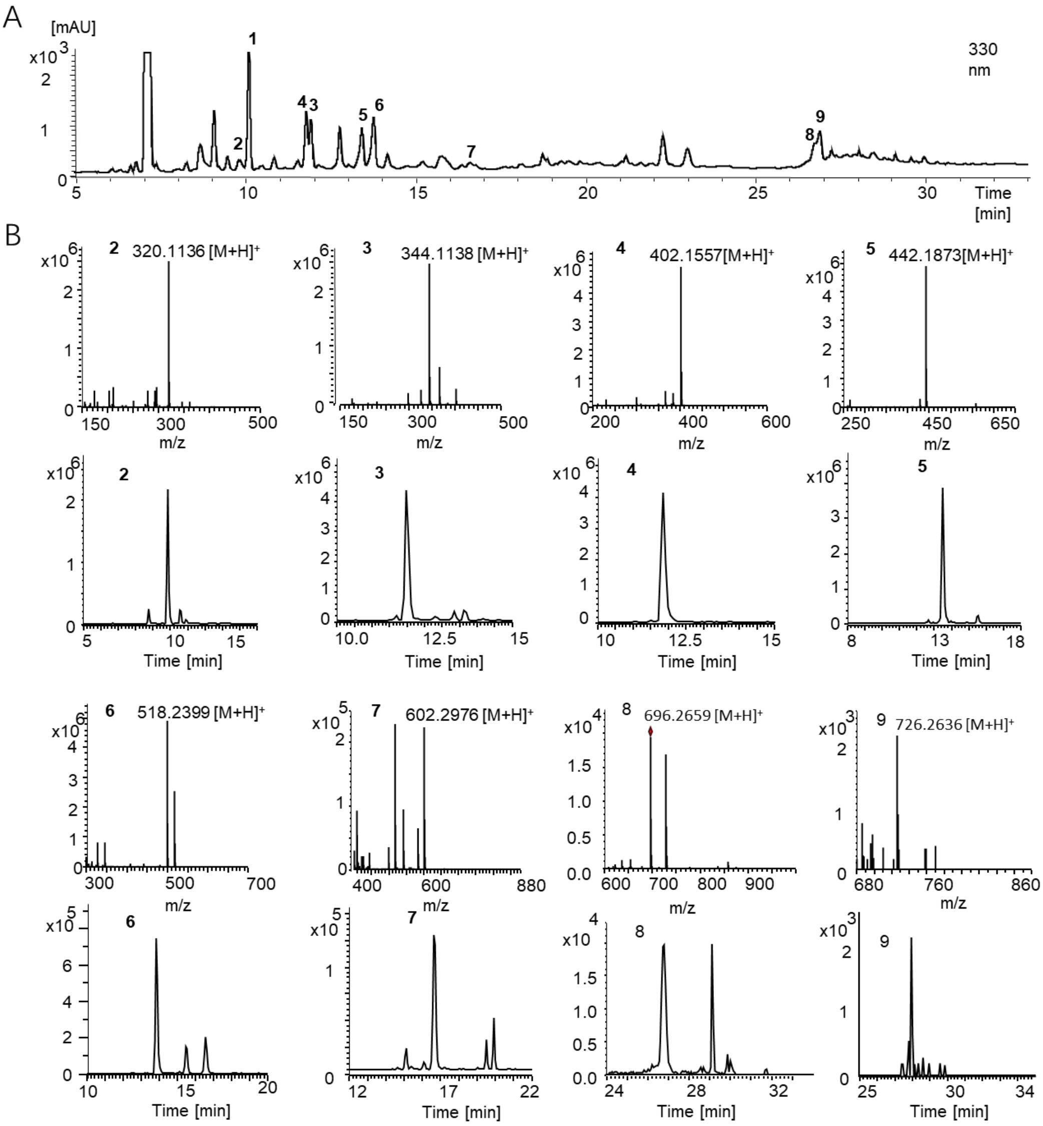

Figure S7. HPLC analysis and HRMS mass map of TRM71106-IL metabolite. (A) HPLC chromatogram of the TRM71106-IL crude metabolite extract; (B) High-resolution mass spectrometry (HRMS) analysis of metabolites **2**–**9** based on m/z values.

References

Kieser T, B. M., Buttner MJ, Chater KF, Hopwood DA. (2000). Practical Streptomyces genetics. *John Innes Foundation, Norwich, United Kingdom.*

Bierman M, Logan R, O'Brien K, et al. Plasmid cloning vectors for the conjugal transfer of DNA from Escherichia coli to Streptomyces spp.. Gene, 1992, 116(1): 43-49.
